# Cotransduction with MGMT and Ubiquitous or Erythroid-Specific GFP Lentiviruses Allows Enrichment of Dual-Positive Hematopoietic Progenitor Cells *In Vivo*

**DOI:** 10.5402/2012/212586

**Published:** 2012-07-19

**Authors:** Justin C. Roth, Mourad Ismail, Jane S. Reese, Karen T. Lingas, Giuliana Ferrari, Stanton L. Gerson

**Affiliations:** ^1^Division of Infectious Diseases, Department of Pediatrics, The University of Alabama at Birmingham, Birmingham, AL 35294, USA; ^2^Division of Hematology Oncology, Case Comprehensive Cancer Center and The Center for Stem Cell and Regenerative Medicine, Cleveland, OH 44106, USA; ^3^H. San Raffaele-Telethon Institute for Gene Therapy (HSR-TIGET), 20132 Milan, Italy

## Abstract

The P140K point mutant of MGMT allows robust hematopoietic stem cell (HSC) enrichment *in vivo*. Thus, dual-gene vectors that couple MGMT and therapeutic gene expression have allowed enrichment of gene-corrected HSCs in animal models. However, expression levels from dual-gene vectors are often reduced for one or both genes. Further, it may be desirable to express selection and therapeutic genes at distinct stages of cell differentiation. In this regard, we evaluated whether hematopoietic cells could be efficiently cotransduced using low MOIs of two separate single-gene lentiviruses, including MGMT for dual-positive cell enrichment. Cotransduction efficiencies were evaluated using a range of MGMT : GFP virus ratios, MOIs, and selection stringencies *in vitro*. Cotransduction was optimal when equal proportions of each virus were used, but low MGMT : GFP virus ratios resulted in the highest proportion of dual-positive cells after selection. This strategy was then evaluated in murine models for *in vivo* selection of HSCs cotransduced with a ubiquitous MGMT expression vector and an erythroid-specific GFP vector. Although the MGMT and GFP expression percentages were variable among engrafted recipients, drug selection enriched MGMT-positive leukocyte and GFP-positive erythroid cell populations. These data demonstrate cotransduction as a mean to rapidly enrich and evaluate therapeutic lentivectors *in vivo*.

## 1. Introduction

 Lentiviral vectors are promising gene transfer agents for *ex vivo* correction of inherited or acquired genetic defects. Hematopoietic disorders are well suited as targets for this approach, due to the relative ease with which these cells are harvested, transduced *ex vivo* with lentivirus, and reinfused into patients. However, even with robust gene transfer efficiencies, the modified cells and their therapeutic potential are diluted upon reinfusion into the large population of endogenous, unmodified cells. Consequently, a survival advantage is needed for transduced cells *in vivo*. However, few inherited hematopoietic disorders, when genetically corrected, provide an inherent survival advantage at the hematopoietic stem cell level. To overcome this limitation, genes that permit selective expansion of transduced cells *in vivo* have been inserted into therapeutic vectors.

**Figure 1:**
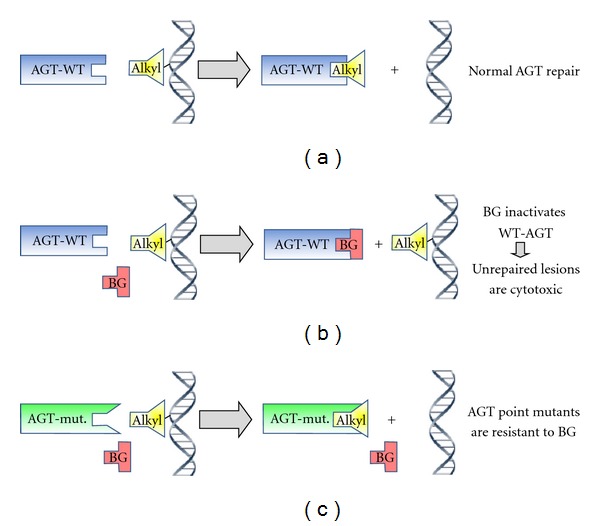
Mutant MGMT-mediated drug selection. MGMT encodes O^6^-alkylguanine-DNA alkyltransferase (AGT). (a) AGT repairs O^6^-alkylguanine lesions in DNA resulting from exposure to methylating or chloroethylating agents, such as, temozolomide and 1,3-Bis(2-Chloroethyl)-Nitrosourea (BCNU), respectively. (b) O^6^-benzylguanine (BG) irreversibly inactivates wild-type AGT, sensitizing cells to O^6^-alkylating agents. (c) Specific AGT point mutations, including P140K and G156A, are resistant to BG inactivation but maintain normal repair activity for O^6^-alkylguanine lesions.

 Several drug resistance genes have been assessed for *in vivo* stem cell selection [1–4]. However, the most stringent *in vivo* stem cell selection results have been achieved with point mutants of O^6^-methylguanine-DNA methyltransferase (MGMT) [5, 6]. MGMT encodes O^6^-alkylguanine-DNA alkyltransferase (AGT). AGT repairs O^6^-alkylguanine DNA lesions induced by methylating and chloroethylating agents, such as, temozolomide and 1,3-Bis(2-Chloroethyl)-Nitrosourea (BCNU), respectively (Figure 1). Specific AGT point mutations, including P140K and G156A, are resistant to the wild-type AGT inhibitor, O^6^-benzylguanine (BG) [7–10]. Inactivation of wild-type AGT with BG sensitizes untransduced bone marrow cells to O^6^-alkylating agents, allowing the resistant MGMT mutant-transduced cells to expand and repopulate [11]. Sustained high-level expression of MGMT has been demonstrated with both single-gene retroviral and lentiviral vectors [5, 6, 12, 13]. Dual-gene vectors containing mutant MGMT have also been shown to efficiently select stem cells *in vivo* [14–16]; however, higher MOIs are often required to compensate for the reduced expression efficiency of one or both genes in dual-gene lentivectors.

 Another strategy utilizes separate single-gene vectors to simultaneously cotransduce cells. This strategy allows two genes to be rapidly evaluated without a need for dual gene vector construction. Cotransduction with separate vectors has been used extensively for *in vitro* models [17, 18], or for derivation of induced pluripotent stem cells [19, 20]. Frimpong and Spectro demonstrated that separate VSV-G-pseudotyped lentiviruses could be used to cotransduce cell lines or primary human neurons. They also demonstrated efficient cotransduction of cells with two bicistronic vectors, each with a unique drug resistance gene, for *in vitro* selection of only dual-positive cells [18].

 The efficiency of hematopoietic cell cotransduction, and whether the cotransduced cells can be enriched *in vivo*, has not been evaluated. Therefore, we evaluated whether separate single-gene lentivectors, one expressing MGMT-P140K, and the other expressing GFP, could efficiently cotransduce hematopoietic cells at low MOIs to produce dual-positive populations that could be enriched with BG and BCNU selection. Cotransduction efficiencies were first assessed in the human erythroleukemia K562 cell line, using various virus ratios, selection stringencies, and total MOIs. This information was then applied to murine bone marrow cell cotransductions to selectively amplify dual-positive cells *in vivo*. Since insertional mutagenesis has been demonstrated as a potential risk associated with gene therapy [21], these studies involved significantly reduced MOIs compared to MOIs reported for *in vivo* selection with dual-gene lentiviral vectors [22–24]. Our data demonstrate that cotransduction, coupled with MGMT-mediated selection, allows enrichment of dual-positive cells *in vivo*. Further, we show that MGMT-mediated HSC enrichment can be coupled to lineage-specific transgene expression from a separate cotransduced lentivector.

## 2. Results

### 2.1. Single Gene Vectors Efficiently Cotransduce Human Hematopoietic Cell Lines

 Selective *in vivo* expansion of hematopoietic cells cotransduced with two separate single-gene lentivectors, one of which provides drug resistance, has not been evaluated. As a first approach to evaluating the efficiency of this strategy, cotransduction and selection were carried out *in vitro* using the human K562 erythroleukemia cell line. A self-inactivating lentiviral vector containing the internal MND promoter [25] was obtained from Donald Kohn (UCLA, Santa Monica, CA). The woodchuck hepatitis virus posttranscriptional regulatory element (PRE) and a multiple cloning site were introduced for subsequent generation of vectors expressing AGT-P140K or the green fluorescence protein (GFP); pMND-MGMT and pMND-GFP, respectively. Both vectors express at high levels in K562 cells, and distinct AGT, GFP, and dual-positive populations can be detected in cotransduced K562 cells.

**Figure 2:**
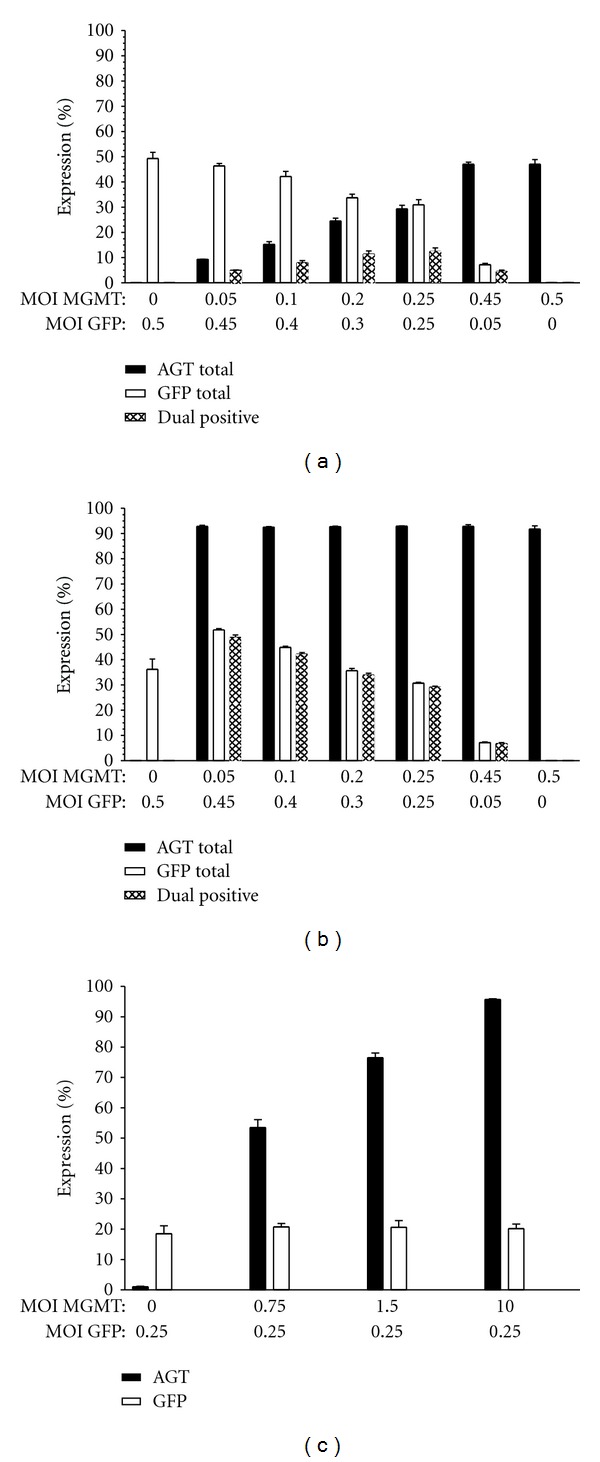
Cotransduction and selective expansion of K562 cells at a constant MOI. K562 cells were cotransduced with varying MGMT : GFP virus ratios with the total MOI held at 0.5. (a) Total AGT, total GFP, and dual-positive percentages in the absence of selection. (b) Treatment with 10 *μ*M BG and 15 *μ*M BCNU enriched both AGT only and AGT-GFP dual-positive populations. Total AGT and total GFP values represent the net single and dual-expression percentages for each gene. The expected level of dual-positive cells is based on the product of the total AGT and total GFP expression percentages for each data point. (c) AGT and GFP expression levels measured separately. Transduction with GFP is unaffected by increased MOIs of the MGMT vector. Error bars indicate ±SEM, *n* = 3.

 The cotransduction efficiency of K562 cells was first evaluated with the total MOI held at 0.5, varying the individual MGMT and GFP virus proportions (Figure 2(a)). The degree of cotransduction was proportional to the total AGT and total GFP expression percentages (“total” refers to both the single-positive and the dual-positive cells). K562 cells cotransduced with an equivalent MGMT and GFP MOI mix (0.25 : 0.25) resulted in the highest AGT^+^ and GFP^+^ total expression percentages. The degree of cotransduction was lowest (4%) when an MGMT : GFP MOI mix of 0.05 : 0.45 was used. However, cells transduced with this MOI mixture had the highest proportion of dual-positive cells (49%) after treatment with 10 *μ*M BG and 15 *μ*M BCNU (Figure 2(b)). Drug treatment enriched AGT^+^ cells to 95% in each culture, with the level of enrichment inversely proportional to the MOI of MGMT used. Both the AGT only and AGT-GFP dual-positive populations were expanded to the same extent, indicating that although both vectors utilize the same promoter, the level of AGT expressed in both populations was equally protective. The percentage of cells expressing GFP did not change after treatment, since the total proportion of GFP^+^ cells in the culture is equivalent to the proportion of GFP^+^ cells expressing AGT. Although GFP expression was maintained in the GFP-only-transduced cultures after drug treatment, survival was markedly reduced and limiting cell numbers were available for analysis. The initially high cotransduction rates achieved using an MOI of 0.25 of each virus had comparatively lower percentages of dual-positive populations after selection (29%), due to the large proportion of MGMT singly transduced cells present in the culture prior to drug treatment. As expected in the setting of drug selection, the highest percentages of dual-positive cells were obtained with virus mixtures composed of low MGMT and high GFP MOI proportions.

 Slightly higher than expected dual-positive percentages were obtained in the K562 cells, compared to calculated values based on the total AGT and GFP expression percentages. To evaluate whether this was the result of transcomplementation of virus proteins during transduction, or the inability to completely resolve the transduced populations, K562 cells were cotransduced with GFP virions at an MOI of 0.25 and MGMT virus MOIs of 0 to 10. The proportion of GFP^+^ cells was then evaluated by flow cytometry in the absence of AGT staining. As shown in Figure 2(c), the transduction efficiency of GFP is independent from the MGMT transduction levels (determined separately by AGT staining), indicating that the slightly elevated cotransduction levels are due to the inability to completely resolve the single- and dual-positive populations by flow cytometry.

**Figure 3:**
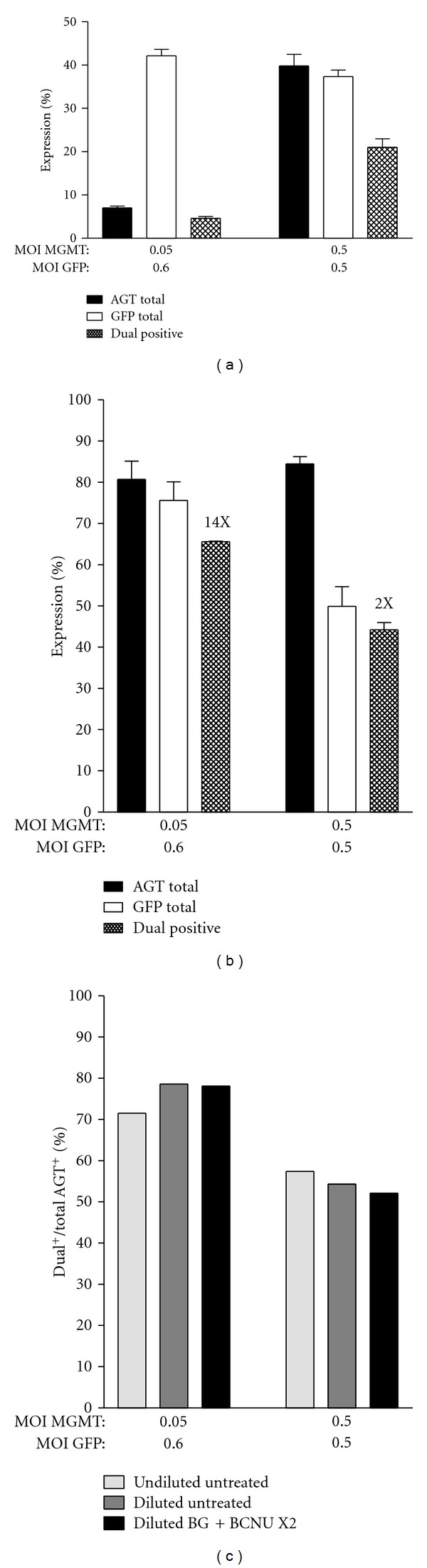
Cotransduction and selective expansion of K562 cells with varying MOIs. K562 cells were cotransduced with a low MGMT : GFP virus ratio or equal amounts of each virus at a higher total MOI. Single- and dual-positive AGT and GFP expression levels were evaluated prior to (a) and after selection with 10 *μ*M BG and 15 *μ*M BCNU (b). Total AGT and total GFP values are the sum of the corresponding single and dual-expressing populations. Fold enrichment of dual-positive populations after drug selection is indicated for each transduction. (c) Cotransduced K562 cultures from panel 3A were undiluted or diluted into a 50-fold excess of untransduced K562 cells and treated twice with 10 *μ*M BG and 15 *μ*M BCNU. The fraction of dual-positive cells in the total AGT-expressing population remains constant after stringent selection conditions. Error bars indicate ±SEM, *n* = 3.

 We next compared the cotransduction efficiency of K562 cells using different total MOIs. Cotransduction of K562 cells with MOIs greater than 1 resulted in a high proportion of dual-positive cells in the absence of selection. However, since high MOIs are associated with an increased risk of insertional mutagenesis, we evaluated whether cotransduction with an optimal MGMT : GFP (0.05 : 0.6) MOI ratio at a low total MOI would result in higher percentages of dual-positive cells, compared to that obtained with a 1 : 1 mixture of the two viruses at a higher total MOI (=1; 0.5 of each virus). Before selection, the level of cotransduction obtained with the highest total MOI was over 4 times that obtained with the 0.05 MGMT and 0.6 GFP virus MOI mix, 21% versus 4.6%, respectively (Figure 3(a)). However, after selection, cultures transduced with the 0.05 MGMT and 0.6 GFP virus MOI mixture had the highest percentage of dual-positive cells (66%), due to the increased rate of dual-positive cell enrichment (Figure 3(b)). These data indicate that selective expansion of cells cotransduced with optimal virus proportions can overcome the reduced transduction rates obtained with low MOIs.

### 2.2. Drug Selection Enriches MGMT-Only and MGMT-GFP Dual-Positive Populations Equally

 Drug selection of cells transduced with dual gene vectors can lead to preferential expression of only the drug resistance marker [26]. To evaluate whether MGMT singly transduced cells out compete dual transduced populations under more stringent expansion conditions, the untreated cotransduction cultures from Figure 3(a) were diluted into a 50-fold excess of untransduced K562 cells and selected with two sequential rounds of 10 *μ*M BG and 15 *μ*M BCNU treatment. As shown in Figure 3(c), the fraction of dual-positive cells in the total AGT^+^ population is equivalent before and after two rounds of drug selection, demonstrating that preferential expansion of MGMT singly expressing cells did not occur.

### 2.3. *In Vitro* Selection Enriches Cotransduced Bone Marrow Progenitor Cells

**Figure 4:**
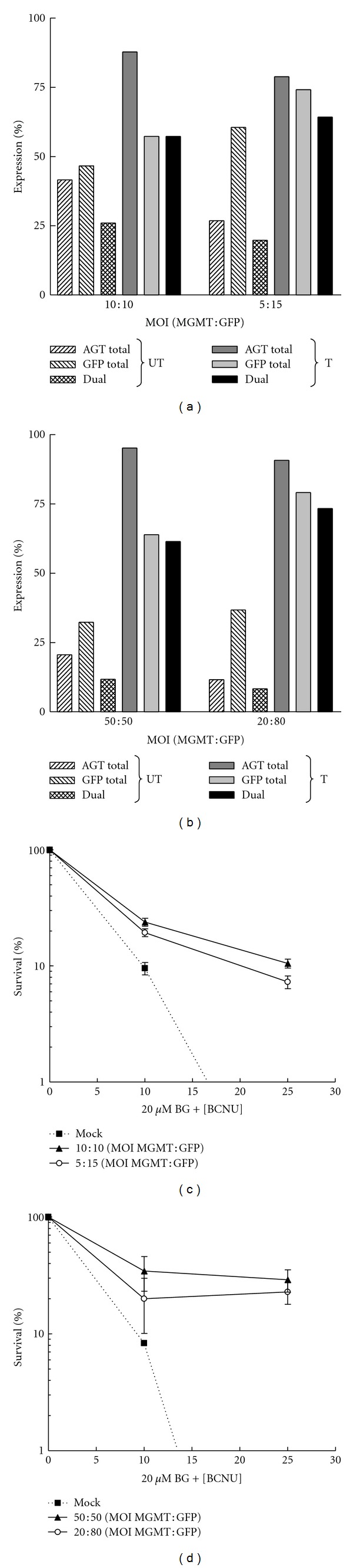
Selective expansion of cotranduced murine bone marrow cells. 5-FU-enriched or SKL cells isolated from whole BM were cotransduced for 12 hours using equal or low MGMT : GFP virus mixtures at equivalent total MOIs. Single- and dual-expression levels in culture-expanded bone marrow (a) or SKL (b) cells before and after drug treatment. The percentage of cotransduced murine bone marrow (c) or SKL-derived, (d) CFU-surviving treatment with 20 *μ*M BG and 0–30 *μ*M BCNU. Error bars indicate ±SEM, *n* = 3.

 We next evaluated the cotransduction efficiency of murine bone marrow (BM) cells using the single-gene MND-MGMT and MND-GFP vectors. Progenitor populations derived from 5-fluorouracil-treated donors, or enriched by Sca^+^/Kit^+^/Lineage^neg^ (SKL) selection, were cotransduced with equal or low MGMT : GFP virus proportions at constant total MOIs. Higher MOIs were used on primary cell cultures to overcome the reduced transduction efficiencies, compared to human cell lines, but well within the range of MOIs used in typical HSC transduction protocols [22–24]. The transduced cells were drug treated and expanded in culture for 10 days to determine AGT and GFP expression levels by flow cytometry (Figures 4(a) and 4(b)) or plated in methylcellulose to calculate the survival percentages of hematopoietic progenitors (Figures 4(c) and 4(d)). Although the MGMT MOIs used to transduce SKL cells were 2–10-fold higher than those used for the BM cultures, the AGT^+^ expression percentages obtained in the unselected SKL populations were half of those observed in the BM cells. Nevertheless, increased progenitor survival percentages were obtained with SKL cells, compared to that of the BM cells, at higher BCNU doses (Figures 4(c) and 4(d)). The dual-positive percentages in both the SKL and BM cultures were much higher than expected values, based on total AGT^+^ and GFP^+^ cell percentages. In addition, the progenitor survival rates obtained with equivalent or staggered MGMT and GFP MOI ratios were not dramatically different in either the SKL or the BM cell assays. These data suggest that progenitor populations that are permissive to transduction at the time of virus addition are efficiently transduced with separate vectors at relatively low MOIs.

### 2.4. Cotransduction Allows *In Vivo* Selection and Lineage Specific Reporter Gene Expression

 Vector size constraints can be a limiting factor for dual-gene vectors. Insertion of two large genes into the same vector can reduce titers and leave little space for other beneficial sequences, such as, posttranscriptional regulatory elements, insulators, or matrix attachment regions [27]. Cotransduction may be particularly applicable to situations in which the transcription of two genes is targeted to different cell types. To evaluate whether codelivery of a ubiquitous MGMT expression vector and a lineage-restricted GFP virus would allow both stem cell selection and expression of GFP in the correct hematopoietic compartment, we used a modified lentiviral GFP reporter vector (pRRL-GATA-GFP) that restricts GFP expression to TER119^+^ murine erythroblasts [28]. Our *in vitro* cotransduction results demonstrated that the reduced transduction efficiency achieved with lower MOIs of the MGMT virus can be circumvented by increased selection stringencies. Thus, as a first approach, SKL cells were cotransduced with an MND-MGMT:GATA-GFP MOI mixture of 5 : 15 and transplanted into 6 lethally irradiated recipients.

**Figure 5:**
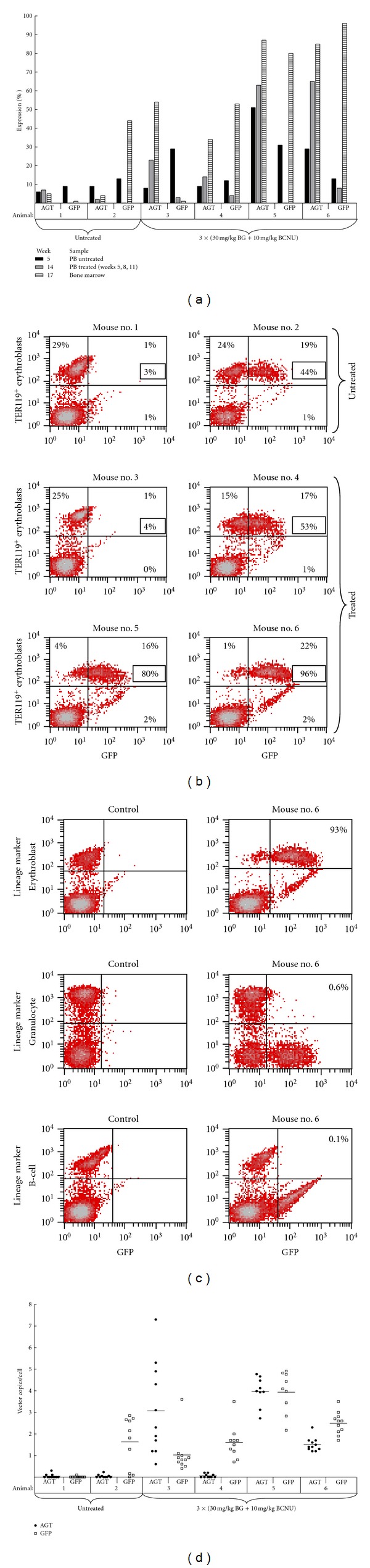
Cotransduction for selection and lineage-specific expression *in vivo*. Donor bone marrow cells were cotransduced with MGMT and RRL-GATA-GFP viruses for 12 hours prior to transplant into lethally irradiated recipients. PB AGT^+^ and TER119^+^-GFP^+^ expression percentages were taken at three week intervals, two days prior to each treatment with 30 mg/kg BG and 10 mg/kg BCNU. (a) AGT^+^ and TER119^+^-GFP^+^ percentages in lethally irradiated mice transplanted with SKL cells cotransduced with an MGMT MOI of 5 and an RRL-GATA-GFP MOI of 15. (b) Flow cytometric analysis of GFP expression in TER119+ bone marrow erythroblasts from animals 1 through 6 in Panel (a) (hatched bars). The percentage positive in each quadrant is listed, as well as the percentage of GFP expression restricted to TER119^+^ erythroblasts (boxed). (c) Expression of RRL-GATA-GFP is restricted to TER119^+^ cells. (d) Analysis of MGMT (“AGT”, closed circles) and GFP (open squares) copy numbers per diploid genome in BM CFU obtained from Panel (a) animals 1 through 6 (hatched bars).

 At five weeks posttransplant AGT^+^ PBMC percentages in the cohort of 6 untreated animals averaged 19% (6–51%), while GFP detection in TER119^+^ erythroblasts averaged 18% (9–29%) (Figure 5(a)). AGT^+^ PBMC averages increased to 41% (14–65%) in the 4 animals selected with three rounds of BG and BCNU treatment, compared to 2–7% in 2 untreated controls. Detection of GFP^+^ cells in TER119^+^ BM mononucleocytes averaged 57% (1–96%) in the 4 drug selected animals, compared to 3–44% in the 2 untreated controls. As expected, GFP expression was restricted to TER119^+^ erythroblasts (Figure 5(b)), and the animals with the highest AGT^+^ cell enrichment also had the highest percentage of GFP^+^ cells in the TER119^+^ BM fraction (Figure 5(c)). Copy number analysis revealed an average of 1–4 copies of each vector per cell (Figure 5(d)). Cotransduction with total MOIs equivalent to that used for dual-gene vectors, results in approximately equivalent vector insertion numbers (data not shown). These data indicate that cotransduction allows efficient lineage-specific gene expression to be coupled to progenitor selection *in vivo*.

**Figure 6:**
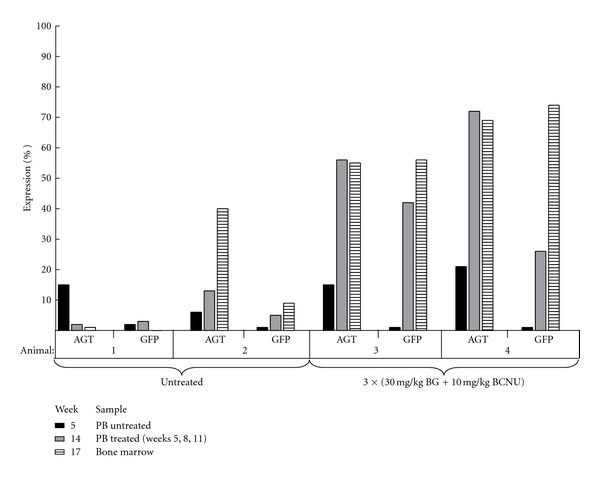
Cotransduction and MGMT-mediated enrichment with limiting MOIs. Whole bone marrow cells from 5-FU-conditioned mice were cotransduced with an MOI of 3 for both MGMT and RRL-GATA-GFP viruses prior to transplant into lethally irradiated recipients. PB AGT^+^ and TER119^+^-GFP^+^ expression percentages were taken at three week intervals, two days prior to each treatment with 30 mg/kg BG, and 10 mg/kg BCNU, as well as in BMMNCs at sacrifice.

 A second transplant experiment was carried out to determine whether this strategy is efficient with limiting MND-MGMT and GATA-GFP MOIs. Whole BM harvested from 5-FU-treated donor mice was transduced with each virus at an MOI of 3. Following a 12-hour transduction, 2 × 10^6^ cells were transplanted into 6 lethally irradiated recipients. At three weeks posttransplant, AGT^+^ PBMC levels in the cohort of 6 untreated animals ranged from 6–27%, while little to no GFP expression was detected (Figure 6). Of the four animals selected with three rounds of 30 mg/kg BG and 10 mg/kg BCNU selection, only two showed evidence of transduced cell engraftment. The AGT^+^ PBMC expression in these two animals was 56–72% after selection, compared to 6–13% in the two untreated controls. In addition, the GFP^+^ percentage detected in TER119^+^ PB erythroblasts was 26–42% in the drug-treated animals, compared to 3–5% in the untreated animals. The mice were sacrificed at 15 weeks posttransplant. The two-drug-treated animals had high percentages of both AGT^+^ mononuclear cells (69% and 55%) and GFP^+^ erythroblasts (74% and 56%, resp.). The number of MGMT and GFP vector insertions in these two animals ranged from 1–3 and 1–4 copies per cell, respectively. These data demonstrate that MGMT-mediated selection permits expansion of cells cotransduced with limiting MOIs and suggests this strategy may serve as first approach to evaluating lineage-specific therapeutic vectors *in vivo*.

## 3. Discussion

 We evaluated whether cotransduction of hematopoietic cells with two separate vectors, one of which allows selection, would provide an efficient alternative to dual-gene vectors. These studies involved significantly reduced MOIs than previously reported for *in vivo* selection with bicistronic lentiviral vectors. We found that 1) the level of cotransduction is proportional to the transduction efficiencies of each vector; 2) at limiting total MOIs, low MGMT : GFP virus proportions resulted in the highest level of dual-positive cells after drug selection; 3) cotransduction allows MGMT-mediated progenitor cell expansion to be coupled to lineage-specific transgene expression. The use of cotransduction for dual-gene delivery eases vector insertion size limits and cis-acting complications that limit the expression efficiency of one or both genes in dual-gene constructs.

 Given that cotransduction involves two genes delivered with separate vectors, we evaluated *in vitro* the stoichiometry of the two viruses used, the total MOI, and the effect of drug selection. When the total MOI was low and held constant, the level of cotransduction in K562 cells was found to be proportional to the transduction efficiency of each vector. The product of fractional proportions is highest when the proportions are equal. Therefore, equivalent levels of each vector resulted in the highest percentage of dual-positive cells in the absence of selection. With drug treatment the initial percentage of GFP^+^ cells is most relevant. The proportion of GFP^+^ cells in the total population is equivalent to the proportion of GFP^+^ cells in the drug-resistant population. Therefore, at a constant MOI, low MGMT : GFP virus ratios resulted in the highest level of dual-positive cells after selection. As previously reported with other vectors [18], the level of cotransduction was slightly higher than the expected values based on the individual transduction efficiencies. However, in the absence of MGMT staining, the transduction efficiency of the GFP lentivirus was unaffected by increasing MOIs of the MGMT virus. Thus the slightly higher dual-positive values seen when AGT and GFP were measured simultaneously are likely due to the inability to completely resolve the single- and dual-expressing populations by flow cytometry.

 The selection stringency must be taken into account when enriching cells that are cotransduced at low MOIs. The degree of expansion obtained with stringent selection conditions can overcome reduced cotransduction rates obtained with limiting MOIs. Low MGMT to GFP virus ratios at a low total MOI resulted in higher levels of dual-positive cells after selection than that obtained with equal mixtures of the two viruses at a higher total MOI. For dual-gene vectors, highly stringent drug treatments have been shown to preferentially select for drug resistance gene expression at the expense of the other gene [26, 29]. However, using a two vector system, MGMT-GFP-transduced cells were enriched to the same extent as MGMT singly transduced cells, even after stringent selection conditions.

 The cotransduction efficiency of primary BM cells is more difficult to predict since it consists of a mixed population of cell types that also differ in their cycling status. Higher MOIs were used in these studies than with human cell lines to overcome the reduced transduction rates obtained with murine BM cells. Although the initial expression percentages were low, the percentage of dual-positive cells obtained after drug treatment was over two times higher than the expected levels calculated from the individual expression percentages. These data indicate that specific cells may be more or less susceptible to transduction and thus, the high MOIs used to increase transduction efficiency of primary cells will likely result in higher integration rates in the susceptible cell populations.

 Cotransduction may have the most potential for situations in which the two genes are transcriptionally targeted to different cell types. This is especially true for severe hemoglobinopathies, including beta-thalassemia major and sickle cell anemia, in which bulky regulatory elements are required for lineage-restricted therapeutic gene expression [30, 31]. We report here the ability to couple MGMT-mediated selection to erythroblast-specific GFP expression using separate single-gene vectors. Selection for engrafted MGMT- and GATA-GFP-positive cells was efficient in both SKL and whole BM transplant recipients.

 Cotransduction represents an alternative to dual gene vectors. This strategy should be useful for evaluating the therapeutic potential of low-copy gene replacement used to correct hematopoietic disease models. Since the gene expression levels are independent with this strategy, a wide range of therapeutic vectors could be rapidly assessed by cotransduction with MGMT. Although cotransduction requires at least two insertions for dual-gene expression, the enhanced expression efficiency achieved with single-gene vectors suggests that fewer total copies may be required to achieve expression levels equivalent to that of dual-gene vectors.

## 4. Methods

### 4.1. Vectors and Virus Production

The self-inactivating lentiviral vector (pCSO-rre-cppt-MCU3-Luc) containing an internal MND promoter [25], and the central polypurine tract/central termination sequence (cPPT/CTS) was kindly provided by D. Kohn (UCLA, Santa Monica, CA).The luciferase gene was removed from this vector by NcoI/EcoRI digestion and replaced with a multiple cloning site cassette. The woodchuck hepatitis virus posttranscriptional regulatory element (obtained from T. Hope) was inserted into the unique EcoRI site. The resulting vector was restricted with NcoI/BamHI for insertion of MGMT-P140K or GFP. The pRRL-GATA-GFP vector was described previously [28]. Virus was generated as described by Zielske et al. [12]. Briefly, 293T cells were triple transfected with the packaging vector (pCMVdeltaR8.91), the VSV-G pseudotyping vector (pMD.G), and either the pMND-MGMT-P140Kpre, pMND-GFPpre, or pRRL-GATA-GFP transfer vectors at a mass ratio of 3 : 1 : 3, using Lipofectamine 2000 (Invitrogen) according to the manufacturer's instructions. Virus produced 24–48 hours after transfection was harvested in Dulbecco's modified Eagle medium (DMEM; Cellgro) containing 10% heat-inactivated FBS (Cellgro) and 2 mM GlutaMAX (Invitrogen). Virus-enriched media was filtered through 0.45 *μ*m syringe filter units (Millipore) and stored at −80°C. Expression titers were determined on K562 cells using virus dilutions that resulted in less than 10% transduction. Titers ranged from 6 × 10^6^ to 3 × 10^7^ expression units/mL.

### 4.2. Cotransductions and Transplants

Virus preps were premixed at the specified MOI ratios prior to the addition of cells. K562 cells were cotransduced in Iscove's medium (Cellgro) containing 10% heat-inactivated FBS, 2 mM GlutaMAX, and 8 *μ*g/mL polybrene (Sigma, St. Louis, MO, USA). Whole BM and sorted stem cell populations were transduced for 12 hrs in alpha-MEM containing 20% heat-inactivated FBS, 2 mM GlutaMAX, 6 *μ*g/mL polybrene, in the presence of 20 ng/mL murine IL-3 and 50 ng/mL of murine IL-6, and SCF (R&D Systems Inc., Minneapolis, MN, USA). The apparent MOIs used for mouse cell transductions were based on expression titers established in K562 cells. The transduced cells were transplanted immediately after transduction. For whole BM transplants, donor marrow, was obtained from 6- to 8-week-old C57Bl/6j mice (Jackson Laboratories, Bar Harbor, ME, USA) 2 days after treatment with 150 mg/Kg 5-FU. Sca^+^Kit^+^lin^−^ (SKL) cells were isolated as previously described [32]. Six-week-old recipient mice were lethally irradiated with 850 cGy using a Cs^137^ source and transplanted with 2 × 10^6^ 5-FU enriched cells per mouse or 1,000 SKL cells supported with 2 × 10^6^ lineage^+^ cells per mouse.

### 4.3. Drug Selection

BG was synthesized by Dr Robert Moschel at the Frederick Cancer Research Institute (Frederick, MD, USA), and BCNU was obtained from the Drug Synthesis and Chemistry Branch of the National Cancer Institute (NCI; Bethesda, MD). All *in vitro* drug treatment incubations were carried out in serum-free media at 37°C. Cells were pretreated with BG for 1 hour, followed by a 2-hour treatment in BCNU. K562 cells were treated in Iscove's media. Drug treatments for *in vitro* murine BM selections were carried out with 25 *μ*M BG and 0, 5, 15, or 30 *μ*M BCNU in alpha-MEM containing 1.2% spleen-cell-conditioned media (Stem Cell Technologies). Drug-treated murine BM samples were expanded 10 days in liquid culture using the same media formulation as described for transduction, without polybrene. Progenitor survival assays were performed as previously described [33]. For *in vivo* selection, BG was dissolved to 3 mg/mL in 40% polyethylene glycol (Union Carbide Corp., Danbury, CT, USA) and 60% PBS (pH 8.0), and BCNU was dissolved in ethanol and diluted to 1 mg/mL in PBS. Mice were injected intraperitoneally with 30 mg/kg BG, followed by 10 mg/kg BCNU an hour later. Three rounds of selection were carried out at 3-week intervals, beginning at 3 weeks for animals transplanted with whole BM and 5 weeks for animals transplanted with SKL cells.

### 4.4. Flow Cytometry

All flow cytometry results were obtained with an LSR flow cytometer (Becton Dickinson). GFP expression was measured in K562 cells after a single wash in PBS. For AGT detection, K562 cells were fixed in 2% paraformaldehyde for 30 minutes at 4°C, permeabilized in 1% Tween-20 for 30 minutes at 37°C, and blocked in 10% normal goat serum for 15 minutes at room temperature. The cells were then immunolabeled with the mouse anti-human MGMT monoclonal antibody MT3.1 (Kamiya Biomedical, Seattle, WA, USA) and an (APC-) conjugated goat anti-mouse secondary antibody (Caltag Laboratories, Burlingame, CA, USA). Dual detection of AGT and GFP expression was carried out with the same staining protocol as that used for AGT alone. Red blood cells were lysed in murine PB and BM samples prior to preparation for flow cytometry.

### 4.5. Copy Number Analysis

Vector insertions per cell were evaluated using a LightCycler instrument and the LightCycler FastStart DNA Master SYBR Green I kit from Roche Diagnostics (Basel, Switzerland). Vector copy numbers were determined using proviral-specific MGMT primers; sense 5′-ACGTCTATATCATGGCCG-3′, antisense 5′-TGAGGATCTTGACAGGATT-3′ and GFP primers; sense 5′-ACGTCTATATCATGGCCG-3′ and Antisense 5′-TGTGATCGCGCTTCTC-3′. Genomic copy numbers were determined using GAPDL4-specific primers, as previously described [12]. MGMT, GFP, and GAPDL4 copy numbers were assessed using a standard curve consisting of murine genomic PBMNC DNA spiked with known vector copy numbers, and verified using K562 cell clones containing a single MGMT-GFP vector insertion. One diploid copy of the mouse genome was assumed to contain two copies of GAPDL4 and 5.9 pg of DNA. Ten CFU were analyzed per animal and the average copy number per CFU was compared to that of total BM from each animal.
